# Melanoma in congenital melanocytic naevi

**DOI:** 10.1111/bjd.15301

**Published:** 2017-04-04

**Authors:** V.A. Kinsler, P. O'Hare, N. Bulstrode, J.E. Calonje, W.K. Chong, D. Hargrave, T. Jacques, D. Lomas, N.J. Sebire, O. Slater

**Affiliations:** ^1^ Paediatric DermatologyGreat Ormond Street Hospital for Children NHS Foundation TrustLondonU.K; ^2^ Paediatric OncologyGreat Ormond Street Hospital for Children NHS Foundation TrustLondonU.K; ^3^ Paediatric Plastic SurgeryGreat Ormond Street Hospital for Children NHS Foundation TrustLondonU.K; ^4^ Paediatric NeuroradiologyGreat Ormond Street Hospital for Children NHS Foundation TrustLondonU.K; ^5^ HistopathologyGreat Ormond Street Hospital for Children NHS Foundation TrustLondonU.K; ^6^ Genetics and Genomic MedicineUCL Institute of Child HealthLondonU.K; ^7^ Developmental Biology and Cancer ProgrammeUCL Institute of Child HealthLondonU.K; ^8^ Dermatopathology DepartmentSt John's Institute of DermatologyGuy's and St Thomas’ HospitalLondonU.K

## Abstract

Congenital melanocytic naevi (CMN) are a known risk factor for melanoma, with the greatest risk currently thought to be in childhood. There has been controversy over the years about the incidence of melanoma, and therefore over the clinical management of CMN, due partly to the difficulties of histological diagnosis and partly to publishing bias towards cases of malignancy. Large cohort studies have demonstrated that melanoma risk in childhood is related to the severity of the congenital phenotype. New understanding of the genetics of CMN offers the possibility of improvement in diagnosis of melanoma, identification of those at highest risk, and new treatment options. We review the world literature and our centre's experience over the last 25 years, including the molecular characteristics of melanoma in these patients and new melanoma incidence and outcome data from our prospective cohort. Management strategies are proposed for presentation of suspected melanoma of the skin and the central nervous system in patients with CMN, including use of oral mitogen‐activated protein kinase kinase inhibitors in *NRAS*‐mutated tumours.

## Abstract 2

**What's already known about this topic?**

- Multiple congenital melanocytic naevi (CMN) are the greatest risk factor for paediatric melanoma.
- Different clinical phenotypes have different risks of malignancy; however, the overall absolute risk for all types of CMN taken together is low.
- CMN can develop proliferative nodules that can cause diagnostic uncertainty and lead to repeated resections.
- Histology in patients with CMN is difficult and often requires specialist review.
- Melanoma in CMN is highly aggressive.

**What does this study add?**

- In our prospective cohort, the strongest statistical risk factor for all‐site melanoma in childhood is an abnormal screening MRI of the central nervous system (CNS) in the first months of life, and in this group the incidence is 12%.
- Where melanoma does arise in children with multiple CMN, a primary in the CNS is at least as common as in the skin.
- CNS melanoma currently has 100% mortality, but oral mitogen‐activated protein kinase kinase inhibition in *NRAS*‐mutation mosaic patients may improve symptom control.
- Management strategies are proposed for the presentation of a possible malignancy in the skin or CNS.

## Congenital melanocytic naevus and congenital melanocytic naevus syndrome

A congenital melanocytic naevus (CMN) is an abnormal but benign collection of naevus cells within the skin at birth. Small single CMN are found in 1% of neonates,^1^, ^2^ where small is defined as < 1·5‐cm projected adult size.^3^ However, there is a spectrum of size and number of CMN, and in around 1 in 20 000 births an infant is born with a naevus of > 20‐cm‐diameter projected adult size,^4^ which is then usually associated with other smaller CMN. In the most severe cutaneous phenotypes, up to 80% of the skin surface area is covered in naevi. The naevi are permanent, and grow in proportion to the child, covering the same anatomical area of skin as is affected at birth.

As with many birthmarks, CMN is the result of a mutation *in utero*, after the embryo has already begun to develop, and which therefore leads to mosaicism. When the mutation occurs early enough in development it can hit a multipotent progenitor cell, which can lead to multiple CMN on the skin and sometimes to involvement of other organ systems. Thus in patients with multiple CMN, the same mutation has been found in different CMN on the skin, and in melanocytic and nonmelanocytic lesions of the central nervous system (CNS), but not in unaffected skin or blood.^5^ Furthermore, such patients can have subtle endocrine dysfunction,^6^ characteristic facial features^7^ and, very rarely, metabolic bone disease, which has so far always been associated with a co‐occurring epidermal naevus.^8^ Where any extracutaneous systems are involved, the term ‘CMN syndrome’ has been proposed^7^ as a more appropriate term than ‘neurocutaneous melanosis’, as melanosis is only a subset of the congenital neurological abnormalities that can occur in patients with CMN. This terminology brings the condition into line with existing classification of other types of congenital naevi.

## Benign proliferations

**Figure 1:**
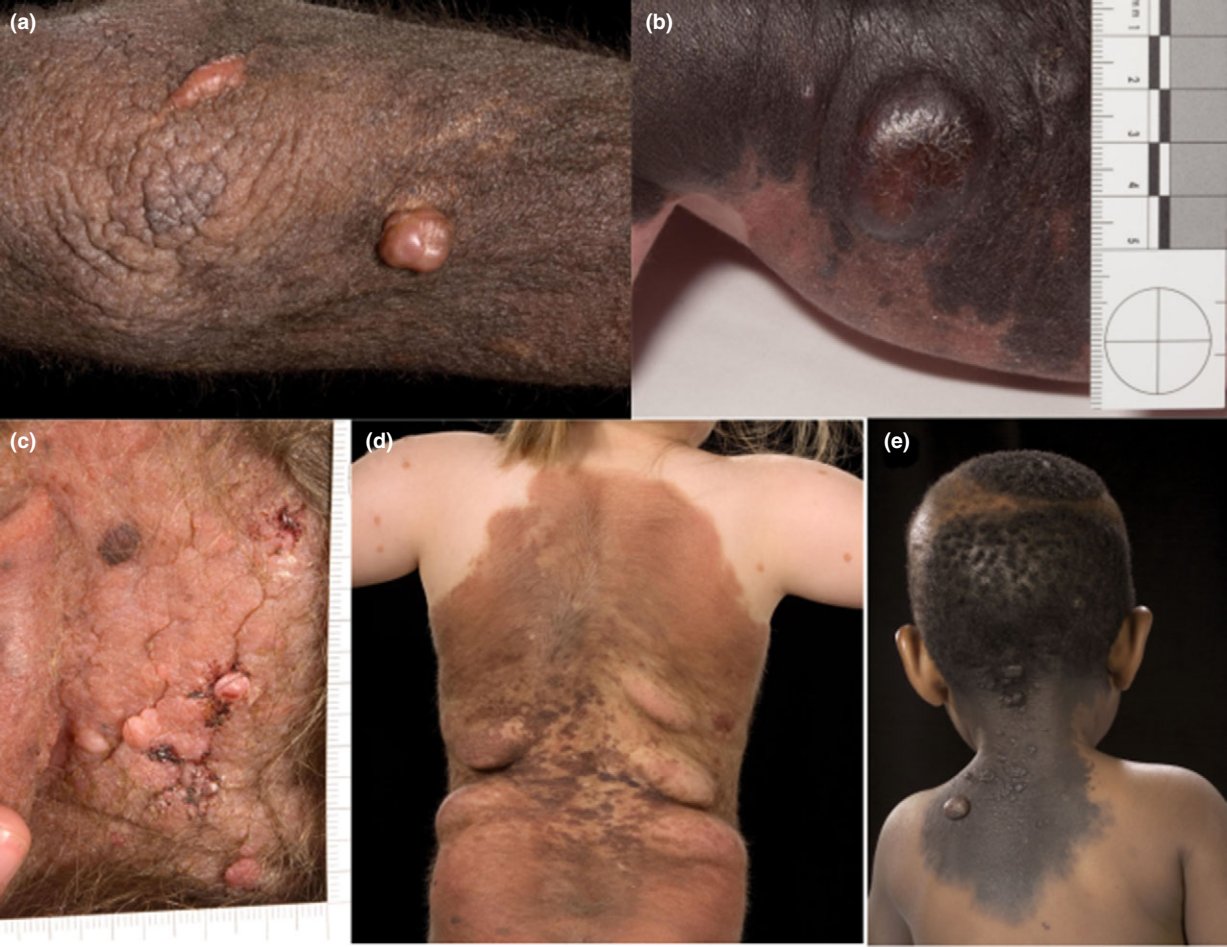
Benign proliferative nodules, which develop commonly within large congenital melanocytic naevi. (a, b, e) Nodules more typical of the ‘classic proliferative nodule’ type; (d) typical ‘neuroid’ type growths; (c) multiple benign proliferations that are not typical of either category. Written consent was obtained for publication.

Benign proliferations within CMN are common, primarily but not exclusively arising in large or multiple naevi, and knowledge of their characteristics is helpful in monitoring for malignancy. Clinical appearances are highly variable (Fig. 1), but the most common can be divided into two categories proposed here: ‘classic’ proliferative nodules and neuroid overgrowth. Classic proliferative nodules have a well‐defined edge, a round or oval outline and a smooth and sometimes shiny surface, and are soft or firm but not hard. They are usually 0·5–2 cm in diameter but can be up to 5 cm, and can be any colour, but are often pink or less darkly pigmented than the surrounding CMN (Fig. 1a, b, e). They are most frequently congenital, but can appear at any time in childhood, when they generally grow over a period of weeks and then stabilize.

**Figure 2:**
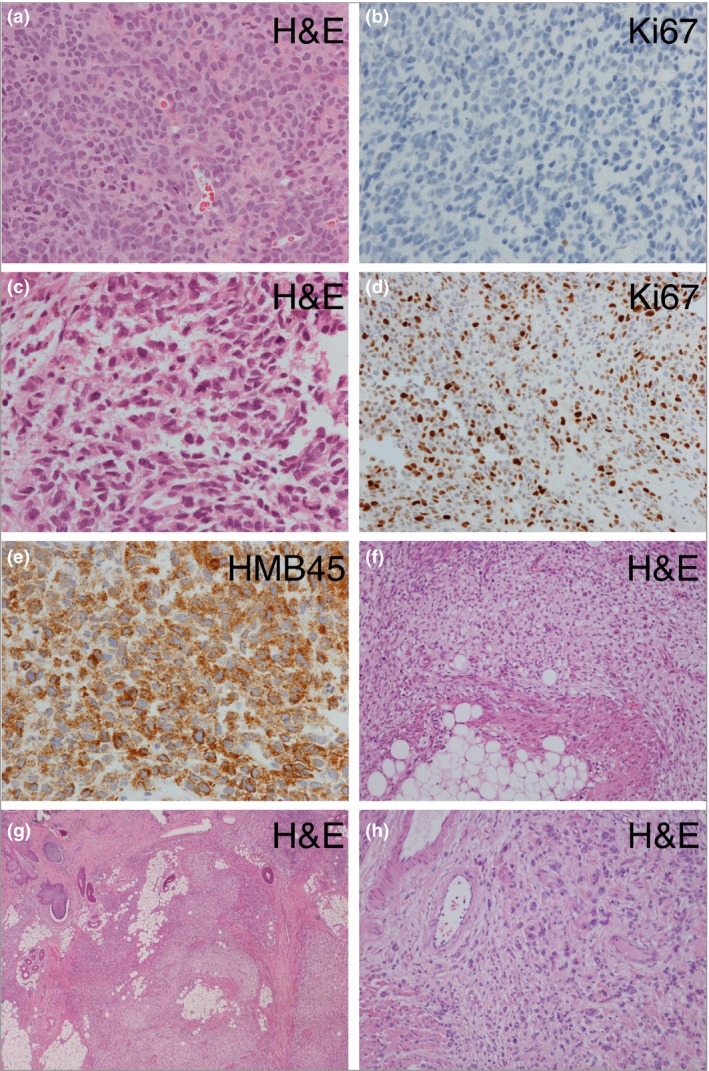
Congenital melanocytic naevus (CMN) – histological features in the nervous system (a–e) and skin (f–h). (a, b) Images of leptomeningeal disease showing a cellular collection of melanocytes with minimal atypia and no significant proliferation, confirmed on Ki67 labelling (b) (patient 3, Table 1). (c–e) In contrast, proliferation of markedly atypical cells with frequent mitotic figures and a high Ki67 labelling index (e). The lesion expresses markers of melanocytes (HMB45). (f–h) Areas in a proliferative nodule within a cutaneous CMN demonstrating typical small deep melanocytes admixed with expansile areas formed of spindled cells and areas with larger cells with eosinophilic cytoplasm; there is no significant atypia and no mitoses are seen. H&E, haematoxylin and eosin.

Histopathology shows a nodular lesion composed of a mixture of morphological cell types with a pushing border, which often distorts the surrounding structures. In contrast to melanoma, there is generally no necrosis, cytological atypia or increased proliferative activity (Fig. 2). If cytological atypia is present, cells are usually homogeneous and mitotic activity is relatively low, although very exceptionally these nodules can be quite proliferative.^9^ Classical proliferative nodules are often suggested to be potential precursors to melanoma; however, convincing documentation of a clearly benign proliferative nodule itself later becoming malignant is difficult to find in the literature,^10^ and has not been seen in our practice. However, in Table 1 we report a case where a benign proliferative nodule was completely resected at birth, and 5 years later a melanoma arose at the same site, suggesting progression, or that some areas may be susceptible to both types of proliferation.

**Table 1:** Clinical and genetic details of 12 patients with congenital melanocytic naevus (CMN) and melanoma seen in our department

Patient number	Sex	Age at diagnosis (years)	Outcome	Screening MRI CNS under 1 year	Primary melanoma site	CMN classification, including recent consensus classification where available^3^	Tissue for genetic investigations	Tissue *NRAS* hotspot genotype (codons 12, 13, 61)	Tissue whole‐genome large (> 1 MB) copy‐number changes
1	Male	Not known	Death, age 7·1 years	Normal	Not known	Multiple CMN, largest > 60 cm PAS. Consensus classification: G2		Not done	Not done
2	Male	~2	Death, age 2·3 years	Not done	CNS, solid tumour in cerebellum	Multiple CMN, no other details available			Not done
3	Female	9·7	Death, age 10·2 years	Normal	CNS, solid tumour in cerebellum, plus diffuse leptomeningeal melanoma, VP shunt, died of spinal cord compression, possible liver metastasis at post mortem	Multiple CMN, largest > 60 cm PAS, bathing trunk, 100–200 naevi in total. Consensus classification: G2, S3, Trunk, C1, R1, N0, H1	Cerebellar melanoma	c.181C>A; p.Q61K	Multiple large gains and losses of whole and parts of chromosomes
4	Male	15·5	Alive 11 months after diagnosis	Normal	Cutaneous, within largest CMN on the back of the scalp and neck, metastatic to local lymph node at time of diagnosis	Multiple CMN, largest 10–20 cm scalp and neck, 10–20 naevi in total. Consensus classification: M2, S1, Neck, C0, R1, N1, H1	Cutaneous melanoma	Wild‐type	This sample tested by FISH: gains 6p25, 11q13
5	Male	1·5	Death, age 2·3 years	Complex congenital neurological disease	CNS, diffuse leptomeningeal melanoma, VP shunt	Multiple CMN, largest PAS > 60 cm, bathing trunk, total naevi 100–200, coexistent X‐linked ichthyosis. Consensus classification: G2, S3, Trunk, C0, R1, N0, H2	Leptomeningeal melanoma	c.181C>A; p.Q61K	Single large duplication of part of 6p
6	Male	0·2	Death, age 3·6 years	Not done	Not known	Multiple CMN, largest PAS > 60 cm. Consensus classification: G2		Not done	Not done
7	Male	Not known	Death, age 2·5 years	Intraparenchymal melanosis only	CNS, diffuse leptomeningeal melanoma, metastasis to peritoneum via VP shunt	Multiple CMN, largest PAS > 60 cm, bathing trunk, 20–50 naevi in total. Consensus classification: G2, S2, Trunk, C2, R2, N2, H1	CMN	c.182A>G; p.Q61R	Not done
8	Male	4·0	Death, age 4·6 years	Complex congenital neurological disease	CNS, diffuse leptomeningeal melanoma, VP shunt	Multiple CMN, largest neck and upper back, cape, PAS 20–40 cm, 100–200 naevi in total. Consensus classification: L1, S3, Trunk, C0, R0, N0, H1	Leptomeningeal melanoma	c.181C>A; p.Q61K	Multiple large gains and losses of parts of chromosomes
9	Female	1·8	Death, age 2·2 years	Complex congenital neurological disease	CNS, diffuse leptomeningeal melanoma, with infiltration of the underlying parenchyma, VP shunt, died of spinal cord compression, no known metastasis	Multiple small CMN, no truly clearly larger naevus although technically one medium CMN, > 400 naevi in total. Consensus classification: S3, C1, R0, N0, H1	Leptomeningeal melanoma	c.181C>A; p.Q61K	Multiple large copy‐number abnormalities
10	Male	Not known	Death, age 2·4 years	Normal	Lymph node groin, locally recurrent despite excision, local metastasis	Multiple CMN, largest PAS > 60 cm, bathing trunk, naevus spilus type (difficult to see and quantify small naevi in this type). Consensus classification: G2, Trunk, C2, R1, N0, H1		Not done	Not done
11	Female	0·2	Death, age 0·9 years	Complex congenital neurological disease	CNS, diffuse leptomeningeal melanoma, VP shunt, died of spinal cord compression, no known metastasis	Multiple CMN, largest PAS > 60 cm, on back, 20–50 naevi in total. Consensus classification: G2, S2	Leptomeningeal melanoma	c.181C>A; p.Q61K	Not done
12	Female	6·5	Death, age 7·1 years	Normal	Cutaneous, within largest CMN, at the site of postnatal resection of a benign congenital nodule, metastatic to local lymph node at time of diagnosis	Multiple CMN, largest on scalp, PAS 10–20 cm, 50–100 naevi in total. Consensus classification: M2, S3, C0, R0, N1, H1	Cutaneous melanoma	c.181C>A; p.Q61K	Gain 1q, 2q, LOH 1p, 5q, 9p, 9q, 11q, 12q, 14q, 17p, 20p

Genotypes of *NRAS* and *BRAF* hotspots and copy‐number changes from tissue biopsies of primary central nervous system (CNS) and cutaneous melanoma are provided where available and consent was given. All patients were wild‐type for hotspots in *BRAF*. For further details of copy‐number changes in the CNS tumours, see Kinsler *et al*.^39^ MRI, magnetic resonance imaging; PAS, projected adult size; VP, ventriculoperitoneal.

Neuroid overgrowth areas have poorly defined edges, are usually round or ovoid/fusiform, are several centimetres to > 20 cm in diameter, can be less pigmented than the surrounding CMN or pink/red, and are soft or lipoma‐like to the touch (Fig. 1d). As a result of their softness they can become pendulous. These are not common at birth, usually developing at any time in childhood and often growing slowly over years. They have a strong predilection for certain areas of the body, in particular the flanks within CMN covering the back and sides, or on the buttocks. Histopathologically these lesions usually show so‐called ‘neuroid differentiation’, being composed of nodules of bland spindle cells within a variably myxoid stroma, without significant cytological atypia or necrosis. A history of transformation to melanoma within this type of lesion has not been reported.

## The genetic basis of congenital melanocytic naevus

### Postzygotic genetics

In single CMN, as with any single lesion, it is difficult to assign a causative mutation. Genes described as mutated in single CMN (or possibly single samples taken from patients with multiple CMN) include *NRAS*,^11^, ^12^ *BRAF*,^11^, ^13^, ^14^, ^15^, ^16^, ^17^, ^18^ *MC1R*,^11^, ^19^ *TP53* 11 and *GNAQ*.^20^ However, in multiple CMN and CMN syndrome it is possible to assign causality to postzygotic mutations in *NRAS* in 80% of cases studied, as the same mutation is found in different cutaneous lesions from the same patient, and in affected neurological and malignant tissue.^5^ Causal mutations in multiple CMN usually lead to amino acid substitutions in codon 61, with p.Q61K being more common than p.Q61R, and with no distinguishable phenotypic differences between these two from existing data. However, numbers of p.Q61R are relatively low and this picture may change.^18^, ^21^ *NRAS* p.Q61H has also been described, but is confined to the rarer naevus spilus phenotypic subtype, a group that also so far contains a single report of a p.G13R mutation^22^ and a p.Q61L.^23^

*BRAF* p.V600E mutations can also be found in individuals with large or multiple CMN^18^ but thus far have not been found in more than one lesion in the same individual, and cannot therefore yet be assigned as causal.

### Germline genetics

Despite the known postzygotic nature of multiple CMN and CMN syndrome, a family history of CMN of any size and number in a first‐ or second‐degree relative has been documented in one‐third of cases in one large cohort.^5^, ^24^ In this same cohort a significant increase in compound heterozygous or homozygous melanocortin‐1 receptor (*MC1R*) variants was discovered in children with CMN, and this was shown to be associated with a positive family history.^19^ Furthermore, certain *MC1R* variants were associated with a more severe cutaneous phenotype.^19^ The mechanism for the interaction between the inherited and postzygotic mutations is not yet understood; however, this pattern mirrors that of sporadic adult melanoma. Whether patients with CMN with germline *MC1R* variants are at an increased risk of melanoma development is not yet known.

### The genetics of proliferative nodules

**Figure 3:**
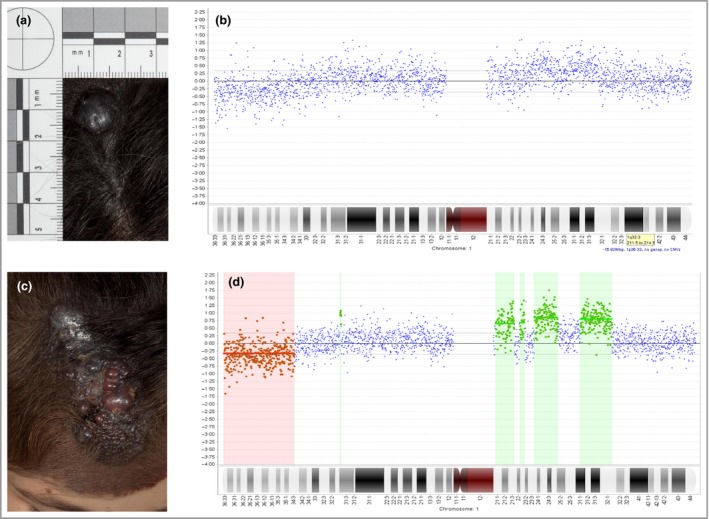
Congenital melanocytic naevus (CMN) – clinical photographs and representative array comparative genomic hybridization traces from chromosome 1 from a new nodule within a scalp CMN that was resected (a, b), but recurred as full‐blown cutaneous melanoma within weeks (c, d) (patient 12 in Table 1). The comparative genomic hybridization data from the nodule demonstrate mosaicism for copy‐number gains and losses, which are then easily seen and called by the program (red and green highlighted areas) in the melanoma sample. The only difference clinically between this nodule and those in Figure 2 was the more rapid rate of growth and failure to stabilize. Written consent was obtained for publication.

It is known to be difficult to differentiate benign proliferative nodules from early melanoma arising within CMN using clinical findings and histopathology alone. Immunohistochemistry of histologically‐benign and atypical proliferative nodules in CMN found that Ki67 and phosphohistone H3 staining have been relatively useful at distinguishing the two, although there was no difference in clinical outcome between the two groups.^20^ Benign and malignant tumours within cutaneous CMN have also been demonstrated to harbour differences in chromosomal copy‐number pattern.^25^ CMN tissue without a proliferative area shows a ‘normal’ pattern of copy‐number changes (i.e. no large gains or losses, where large is defined as > 1 MB), benign proliferative nodules typically show copy‐number changes involving whole chromosomes only, and melanoma typically shows multiple large gains and losses of parts of chromosomes (Fig. 3). This clear distinction in copy‐number patterns with benign and malignant behaviour from this first study has not always been replicated in other studies, with both histopathologically and clinically benign nodules occasionally exhibiting regional rather than whole chromosome copy‐number changes, and clinically and histopathologically malignant nodules the opposite.^26^, ^27^ As with immunohistochemical studies, copy‐number measurement can therefore be seen as a very useful adjunct to other assessment, rather than a definitive test of malignancy.

## Melanoma in congenital melanocytic naevus

### Melanoma incidence and presentation

Patients with CMN have long been known to be at risk of melanoma. Here we will review the evolution of the understanding of this risk, including our own latest prospective cohort data, analysed with respect to different aspects of the congenital phenotype.

Early estimates of melanoma risk were inaccurate due to small study size, the retrospective (often post mortem) nature of the analyses,^28^ and confusion over terminology for CNS disease. Prospective studies of larger groups and systematic literature reviews have now established that the incidence of melanoma is low, of the order of 1–2%.^28^, ^29^, ^30^, ^31^ However, this is an average figure for all CMN and the incidence actually varies enormously with the severity of the congenital phenotype.^28^, ^29^ The risk for small single CMN is very low,^28^, ^29^ whereas where the largest CMN is > 40 cm projected adult size, and accompanied by multiple smaller CMN, the lifetime risk has been estimated at 10–15%.^28^, ^29^ A further complication is that in a substantial proportion of cases the primary melanoma develops within the CNS rather than the skin.^24^, ^28^, ^32^ A recent review of the literature suggests that primary CNS melanoma accounts for approximately one‐third of melanoma occurring in patients with CMN;^32^ however, the exact risk is difficult to quantify due to historical difficulties in differentiating congenital neurological abnormalities from CNS melanoma, and from the historical assumption that the presentation of CNS melanoma must have been a secondary from an unknown primary in the skin.

Long‐term prospective studies in our cohort have found CNS melanoma to be more common in childhood than cutaneous melanoma, (Table 1).^33^ In this cohort of 450 patients there have been 12 cases of melanoma; however, for two of these there is uncertainty whether they were referred before melanoma development, so they were excluded from all incidence calculations. This reduces the cohort to 448, and clinical phenotyping data for this cohort are given in Appendix S1 (see Supporting Information). In the whole group the incidence of melanoma in childhood (0–16 years) is 2·2%, with a mean and median age at death from melanoma of 3·9 and 2·5 years, respectively. All 10 cases were in children with multiple CMN (two or more at birth), and seven of 10 cases occurred in the group where the largest CMN was > 60 cm projected adult size or where there were multiple CMN with no large naevus. This gives an incidence of melanoma in children with CMN > 60 cm projected adult size of 8%, and an incidence of 1% in those who are in any other cutaneous phenotypic group (Table 2). Reliable data on the total number of naevi at first presentation were available in only seven cases, and in four of seven they had > 50 naevi in total. It is worth noting that the patients with CMN in our tertiary centre have phenotypically more severe disease than in the spectrum of CMN seen in the general population.

**Table 2:** Most recent analysis of incidence of melanoma in children (age 0–16 years) with congenital melanocytic naevus (CMN) by different phenotypic groupings

Phenotypic subdivisions of the same cohort	*n*/*N* (%) of cutaneous melanoma in childhood	*n*/*N* (%) of primary CNS melanoma in childhood	*n*/*N* (%) of melanoma with lymph node (*n *=* *1) or missing data primary site (*n *=* *2)	Total *n*/*N* (%) of melanoma in childhood
Single CMN of any size	0/82 (0)	0/82 (0)	0/82 (0)	0/82 (0)
Multiple CMN where the largest CMN is < 60 cm projected adult size	2/199 (1)	0/199 (0)	1/199 (< 1)	3/199 (1)
Multiple CMN where the largest CMN is > 60 cm projected adult size, or where there is no one clearly larger CMN	0/88 (0)	6/88 (7)	1/88 (1)	7/88 (8)
Multiple CMN of any size or number and a normal screening MRI of the CNS	1/179 (< 1)	1/179 (< 1)	1/179 (0)	3/179 (2)
Multiple CMN of any size or number, and the finding of any congenital neurological disease seen on screening MRI of the CNS	1/51 (2)	5/51 (10)	0/51 (4)	6/51 (12)

Data collected prospectively from our U.K. cohort, 1988–2016, where face‐to‐face phenotyping occurred and melanoma was not present at the time of referral (*n *=* *448). Ten children developed melanoma in this prospective cohort, of whom nine had had a screening magnetic resonance imaging (MRI) of the central nervous system (CNS). Multiple CMN is defined as two or more at birth. Where numbers do not add up to 448 this is because of individual items of missing data in the older phenotyping data.

However, recent data have shown that the risk of melanoma appears to be higher in those with congenital abnormalities of the CNS.^33^ In line with this, melanoma incidence in the group of multiple CMN with an abnormal screening magnetic resonance imaging (MRI) of the CNS in the first year of life was still higher, at 12%, whereas in those with a normal screening scan it was 1–2% (Table 2). In logistic regression modelling of any‐site melanoma, an abnormal screening MRI of the CNS in the first year of life was the strongest statistical predictor, better than any clinical phenotyping variable (odds ratio of all‐site melanoma with an abnormal MRI 16·7, 95% confidence interval 3·0–92·3, *P *=* *0·001, when the model was corrected for projected adult size). CNS screening MRI is therefore currently the best predictor of all adverse outcomes in children, with those with a normal scan being in a low‐risk group for all complications, independent of the rest of their clinical phenotype.

It is not yet clear why there is such a strong association between screening CNS MRI results and overall risk of melanoma. It could be because CNS melanoma is more common in our cohort and that this is the real association with abnormal CNS MRI rather than all‐site melanoma. Other possible explanations are that abnormal MRI is an indicator of a higher burden of mutated cells in the body as a whole, or that the mutation in those with complex congenital neurological disease happened at a particular stage of development, or that those with an abnormal MRI have other genetic risk factors predisposing both to congenital neurological disease and malignancy.

Cutaneous melanoma arising in CMN usually presents as a new nodule or lump,^32^, ^34^ arising mainly in the deeper dermis or subcutis, and generally with a high Breslow thickness at presentation.^32^ These features were found in the cases of cutaneous melanoma arising in our prospective cohort (Table 1), where local lymphadenopathy due to metastasis was also a presenting feature.

Primary CNS melanoma in individuals with multiple CMN can present in different ways. These are either as a solid tumour within the brain parenchyma, or more commonly as leptomeningeal melanoma, a diffuse and rapidly progressive proliferation of melanin‐producing cells within the leptomeninges. These two can exist separately, but where they coexist they can be physically unconnected,^35^ or the leptomeningeal melanoma can invade the underlying parenchyma.^36^ Patients can present with focal neurological symptoms such as seizures, and/or with signs and symptoms of raised intracranial pressure.^33^, ^37^, ^38^ This is secondary to diffuse melanocytic leptomeningeal disease, which may not be evident on MRI at the time of presentation, although hydrocephalus usually is; in these cases we suggest that a repeat MRI should be performed after 2 weeks if symptoms persist, and the leptomeningeal disease is usually then detectable. Very rarely‐described is diffuse leptomeningeal disease that stabilizes and does not progress to death.^33^, ^39^ Stable focal leptomeningeal deposits are also described.^33^, ^38^

The histopathological examination of all of these leptomeningeal lesions can be challenging. Biopsies show cellular collections of melanocytes, which may show a variety of morphological features but typically have minimal atypia or proliferation, and no invasion of CNS parenchyma (Fig. 2). Transformation to malignancy is defined histologically by unequivocal invasion of the CNS parenchyma, and/or cytological atypia and cellular proliferation (Fig. 2). However, the prognosis is often poor despite the lack of classical cytological hallmarks of malignancy. The whole clinical picture should therefore be taken together, and a very high index of suspicion maintained.

Melanoma has very rarely been described in sites other than the skin or CNS, specifically the lymph nodes^24^ and the retroperitoneum.^40^ Children with CMN can also rarely develop tumours other than melanoma, most commonly rhabdomyosarcoma.

### The genetics of melanoma in congenital melanocytic naevus

It has been demonstrated that further mutations are required to cause malignant transformation in a CMN. Those described relating to *NRAS* are loss of the normal allele in *NRAS*,^5^ in that case not secondary to a deletion and therefore probably due to postmitotic recombination, and amplification of mutant *NRAS*.^41^ Mutations in *BRAF* have not been described in melanoma arising in a patient with CMN; however, given the availability and efficacy of BRAF inhibitors it is suggested that both *NRAS* and *BRAF* hotspots should be genotyped in cases of melanoma.

Copy‐number measurement is a well‐established test to aid melanoma diagnosis,^42^, ^43^ and changes in cutaneous melanoma arising in CMN are described above. Recent data have demonstrated that the same pattern of changes is also seen in CNS melanoma in patients with CMN,^35^ namely large gains and/or losses of parts of or whole chromosomes. This has confirmed that new or rapidly progressive CNS disease in patients with CMN, often previously termed ‘symptomatic neurocutaneous melanosis’, is, as interpreted by many experts in the field, primary melanoma of the CNS. This therefore is a clinically useful test as an adjunct to clinical, radiological and histopathological assessment in all types of suspected melanoma arising in patients with CMN.

### Melanoma monitoring

Given the rarity of cutaneous melanoma in single CMN of any size routine monitoring is not recommended. In addition, at the moment, there is no evidence that clinical monitoring for cutaneous melanoma in children with multiple CMN or CMN syndrome makes any difference to outcome, and abandoning routine monitoring is arguably reasonable for either physicians or patients. This is partly due to the difficulty in treatment of melanoma in this condition, which in itself is partly due to the difficulty in detection of melanoma within very large CMN. However, regular contact with patients with multiple CMN is often required in childhood for skincare, neurodevelopmental follow‐up, coordination of psychological support, treatment of pruritus or superficial infections where they arise, resection of small CMN where it can clearly improve cosmetic appearance, and to some degree reassurance of contact with a doctor in case it is needed. The basis of skin monitoring with many and extensive naevi is high‐quality photography. Most children with multiple CMN have lesions that are too large and too numerous to be visualized systematically by dermoscopy; however, mole mapping or monitoring by photography on a semiautomated basis may be helpful to look for changes. For smaller CMN, dermoscopic features have been well delineated.^44^

The picture with regards to monitoring for CNS melanoma is changing. In the same way as a patient with benign CMN at birth can develop a cutaneous melanoma, so there can be congenital CNS disease that is benign and stable, or the patient can develop a CNS melanoma. This distinction has not historically been made very clear in the literature, with all types of CNS disease being grouped under the term ‘neurocutaneous melanosis’, with some effort to differentiate different types of disease by using the term ‘symptomatic neurocutaneous melanosis’. This is an inappropriate term as many children with benign stable melanotic disease of the CNS have very substantial symptoms such as neurodevelopmental delay or seizures, but do not have a poor prognosis with respect to life expectancy.^33^, ^38^

Monitoring for CNS melanoma ideally requires a single screening MRI to characterize the congenital disease in the CNS if any, and therefore to act as a baseline should the child present with new neurological symptoms at any stage in life. For this we recommend a brain and whole‐spine MRI with gadolinium contrast under the age of 1 year (under 6 months ideally) for anyone with two CMN at birth independently of size or site.^29^, ^33^ This can be considered comparable with a proper examination and documentation of the skin. This recommendation is made on the basis that in our institution we do not routinely use general anaesthesia for this procedure under the age of 1 year. However, even if this is not an option the scan is pivotal in giving an accurate prognosis, and radically alters clinical management.

The rationale for scanning at all is to stratify the management of patients, as the scan result has been shown to be the best statistical predictor of clinical outcomes,^33^ including melanoma (Table 2). The rationale for scanning under the age of 6 months is for best visualization of the characteristic signal for melanin under MRI, before full myelination takes place. If normal, the screening scan does not need to be repeated routinely, and no other CNS monitoring is specifically required, and as this applies to 80% of cases is highly reassuring for both clinicians and the families. If the MRI demonstrates the relatively common finding of intraparenchymal melanosis, imaging does not require repeating routinely, but we suggest that annual neurodevelopmental monitoring should be undertaken until school age as there is an increased incidence of neurodevelopmental problems and seizures in this group.^33^ This annual monitoring should allow the early detection of developmental issues (for example speech delay) and the implementation of the best care package for the child (for example speech therapy).

In the small group (<10%) where the MRI demonstrates any other abnormality, regular clinical and/or radiological monitoring is advised, as there is a high incidence of neurodevelopmental abnormalities and seizures and a high rate of needing neurosurgery, and this group appears to be at highest risk of melanoma (Table 2).^33^ Clinical and radiological monitoring should be designed on an individual basis, as this is a very heterogeneous group. Repeat MRI would be recommended in all cases of leptomeningeal disease until the clinical and radiological appearances are definitely stable, as this presentation is known to evolve into melanoma in many reported cases.^33^, ^38^

Independent of the initial screening MRI findings, all families of children with multiple CMN should be made aware that they should present promptly to a clinician if the child develops signs of raised intracranial pressure at any age, or develops new cutaneous lumps or other changes.

Historically all decisions regarding melanoma monitoring in CMN have been based on the severity of the cutaneous phenotype, with significant variation in worldwide practice, and have been established through experience and a small number of cohort studies. New clinical classifications that include not only the size and number of lesions but also colour, heterogeneity and rugosity^3^ may be able to refine this still further going forward. The recent introduction of routine genotyping for germline *MC1R* variants and somatic *NRAS* and *BRAF* mutations may also help to identify individuals at highest risk of melanoma development in the future.

### Melanoma diagnosis

Where the diagnosis of cutaneous melanoma arising in a CMN is suspected clinically, an urgent biopsy should be performed (excision if possible), with histopathological examination by at least two experts. *NRAS* and *BRAF* hotspot genotyping by sensitive methods, and array comparative genomic hybridization (CGH)–single‐nucleotide polymorphism (SNP) array or fluorescence *in situ* hybridization (FISH) for copy number are recommended to improve diagnostic accuracy and to guide management. Driver mutation genotyping is a routine part of assessment of melanoma as this leads to treatment stratification with targeted therapies. This holds true in the context of CMN genotyping, particularly as the majority of melanomas will be *NRAS* mutated, and BRAF inhibitors are currently contraindicated in *NRAS*‐mutated melanoma due to known paradoxical activation of RAS by these drugs.^45^ In addition, second‐hit changes in *NRAS* have been detected in melanoma in CMN, including loss of the normal allele^5^ and copy‐number amplification.^41^ Where the diagnosis of melanoma arising within the CNS is suspected, an urgent MRI of the brain and whole spine with and without contrast enhancement should be performed, and ideally compared with the screening MRI undertaken in the first 6 months of life. Evidence of new suspicious CNS lesions at any age should be investigated as for melanoma by fresh‐tissue biopsy from the CNS. Sampling of cerebrospinal fluid is not recommended routinely as a biopsy is superior, but if a biopsy is not possible for some reason then it may be contributory, and histology of cerebrospinal fluid has been described.^36^

### Melanoma management

**Figure 4:**
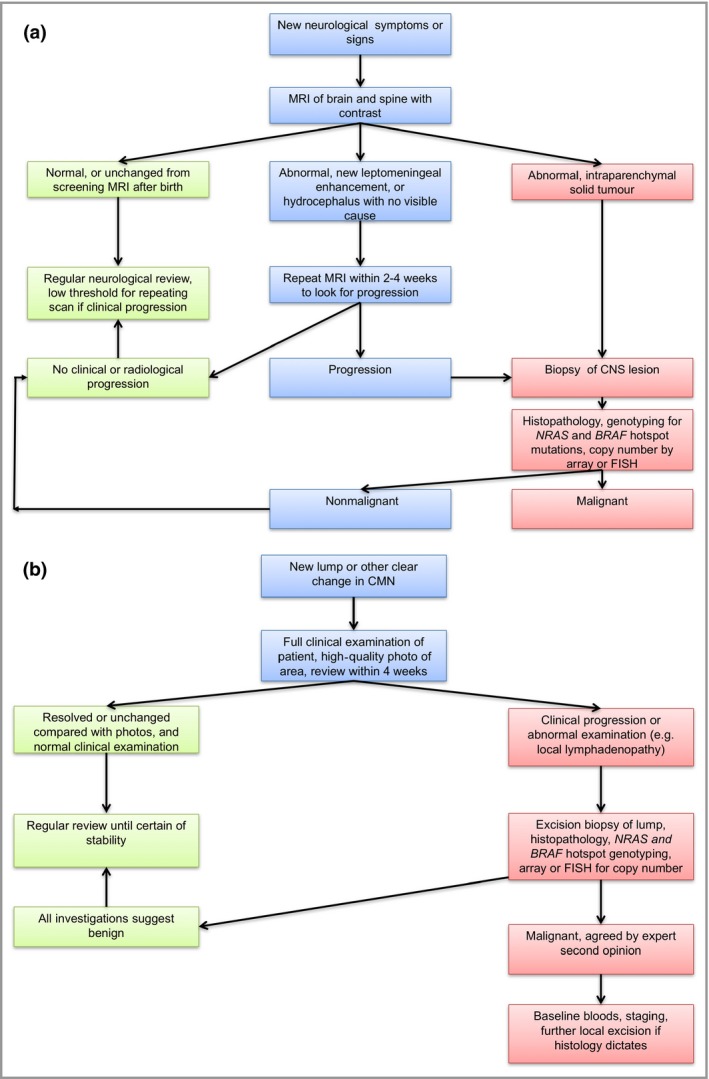
Congenital melanocytic naevus (CMN) – management pathways for suspected malignancy. (a) Proposed clinical pathways for investigation of a patient with CMN with new neurological symptoms or signs [possible central nervous system (CNS) melanoma]. (b) Proposed management of a new lump arising in a CMN. 4/52, 4 weeks; CGH, comparative genomic hybridization; FISH, fluorescence *in situ* hybridization; LDH, lactate dehydrogenase; MRI, magnetic resonance imaging; PET, positron emission tomography.

There are no guidelines for the treatment of melanoma arising in CMN, or in the CNS of patients with CMN. What is offered here is a distillation of multidisciplinary experience in one tertiary centre with a special interest in the condition, and these are suggested guidelines only. Suggested management for a new neurological presentation and for a new lump are detailed in Figure 4. Routine surgical excision of CMN is not part of our management as there is no evidence that it alters melanoma risk, and those with completely excisable CMN and no CNS disease are at very low risk.

Once a diagnosis of melanoma is confirmed, baseline staging investigations should be performed. In most cases of CNS melanoma, distant metastases do not exist at the time of presentation or appear to play a role before death from spinal cord compression. However, in cutaneous melanoma, metastasis to local lymph nodes does occur, and local recurrence within resection sites is rapid even where excision is reported as histologically complete.

Due to the aggressive nature of melanoma in the context of CMN, surgical excision plays an important but usually not a curative role. For cutaneous melanoma this includes excision biopsy if possible, or biopsy for confirmation of diagnosis, with subsequent wide local excision and removal of local nodal metastases. Leptomeningeal melanoma within the spinal canal is typically diffuse and circumferential precluding therapeutic surgical decompression; however, a ventriculoperitoneal shunting procedure is key in symptom management. As the disease progresses, dexamethasone can be useful in the short term for alleviation of raised intracranial pressure. In late stages palliative radiotherapy has been found to be useful in our patients both in leptomeningeal melanoma and in cutaneous melanoma to decrease the bulk of disease temporarily.

Animal model data of mitogen‐activated protein kinase kinase (MEK) inhibitor use has recently demonstrated an attenuation of leptomeningeal disease in a murine model of CMN syndrome.^46^ As a result we have used the MEK inhibitor trametinib as therapy in a small series of four patients with *NRAS*‐mutated CNS melanoma (three of whom had CMN).^47^ This demonstrated measurable and objective relief of symptoms and signs in all patients, although to varying degrees. Further treatments will be needed to address the inevitable progression of melanoma in these patients.

Suggested work‐up for a patient with CMN and a confirmed diagnosis of melanoma is the following. (i) Bloods: full blood count, urea and electrolytes, liver function tests, lactate dehydrogenase, lipid profile, vitamin D level and bone profile, thyroid function, creatine kinase, glycated haemoglobin level, total protein and glucose. (ii) Imaging: CNS MRI with gadolinium contrast, whole‐body positron emission tomography–computed tomography scan, echocardiogram, electrocardiogram and plain radiograph of the wrist and tibial growth plate. (iii) Tissue sample: biopsy of suspected primary (CNS including leptomeningeal, or skin), for histopathology, *NRAS* and *BRAF* hotspot genotyping, copy‐number analysis (array CGH or SNP array or FISH). (iv) Other: ophthalmology assessment, urinalysis.

## Conclusions

Small single CMN are common birthmarks with very low risk of melanoma, and do not require routine resection for this reason. Multiple CMN (two or more, of any size or site) can have extracutaneous associations, then termed ‘CMN syndrome’, and these phenotypes are caused by postzygotic mosaicism for *NRAS* mutations in 80% of cases. Individuals with multiple CMN do have an increased risk of melanoma, particularly in the presence of congenital neurological abnormalities on screening MRI in the first 6 months of life. Melanoma can arise either in the skin or as a primary in the brain, the latter being more common in our prospective study of affected children, or very rarely in other sites. All forms are usually highly aggressive and fatal. Histopathology by at least two experts in the field, plus genetic analysis of driver mutations and copy number can help to differentiate melanoma from benign proliferative nodules in the skin, or from stable congenital disease in the CNS. Treatment with MEK inhibition on a compassionate basis has shown substantial although temporary signs of symptomatic improvement of disabling neurological symptoms in a small series of patients with CNS melanoma.

## Supporting information


**Appendix S1.** Clinical phenotyping statistics for this prospectively recruited cohort of 448 patients with congenital melanocytic naevi.
