# Supplementary material for: Melanoma in congenital melanocytic naevi
Source: Br J Dermatol. 2017 Apr 4;176(5):1131–43. doi: 10.1111/bjd.15301 (PMC5484991; doi:10.1111/bjd.15301)
Supplement: Supplementary file 1 — Appendix S1. Clinical phenotyping statistics for this prospectively recruited cohort of 448 patients with congenital melanocytic naevi. [file BJD-176-1131-s001.docx]

**Supplementary data**

Clinical phenotyping statistics for this prospectively recruited CMN cohort of 448 patients

| **Sex** | | | | | |
| --- | --- | --- | --- | --- | --- |
|  | | Frequency | Percent | Valid Percent | Cumulative Percent |
| Valid | female | 244 | 54.5 | 54.6 | 54.6 |
|  | male | 203 | 45.3 | 45.4 | 100.0 |
|  | Total | 447 | 99.8 | 100.0 |  |
| Missing | System | 1 | .2 |  |  |
| **Total** | | 448 | 100.0 |  |  |

| **Largest CMN Projected Adult Size** | | | | | |
| --- | --- | --- | --- | --- | --- |
|  | | Frequency | Percent | Valid Percent | Cumulative Percent |
| Valid | <10cm | 106 | 23.7 | 24.3 | 24.3 |
|  | 10-20cm | 90 | 20.1 | 20.6 | 44.9 |
|  | 20-40cm | 86 | 19.2 | 19.7 | 64.5 |
|  | 40-60cm | 60 | 13.4 | 13.7 | 78.3 |
|  | >60cm | 83 | 18.5 | 19.0 | 97.3 |
|  | No clearly larger CMN (multiple small or multiple medium) | 12 | 2.7 | 2.7 | 100.0 |
|  | Total | 437 | 97.5 | 100.0 |  |
| Missing | System | 11 | 2.5 |  |  |
| Total | | 448 | 100.0 |  |  |

| **Multiple CMN at birth (two or more)** | | | | | |
| --- | --- | --- | --- | --- | --- |
|  | | Frequency | Percent | Valid Percent | Cumulative Percent |
| Valid | no | 84 | 18.8 | 22.6 | 22.6 |
|  | yes | 287 | 64.1 | 77.4 | 100.0 |
|  | Total | 371 | 82.8 | 100.0 |  |
| Missing | System | 77 | 17.2 |  |  |
| **Total** | | 448 | 100.0 |  |  |

| **Approximate total number of naevi at birth** | | | | | |
| --- | --- | --- | --- | --- | --- |
|  | | Frequency | Percent | Valid Percent | Cumulative Percent |
| Valid | one | 62 | 13.8 | 21.5 | 21.5 |
|  | 2-9 | 86 | 19.2 | 29.9 | 51.4 |
|  | 10-20 | 54 | 12.1 | 18.8 | 70.1 |
|  | 20-50 | 46 | 10.3 | 16.0 | 86.1 |
|  | 50-100 | 22 | 4.9 | 7.6 | 93.8 |
|  | 100-200 | 10 | 2.2 | 3.5 | 97.2 |
|  | >200 | 7 | 1.6 | 2.4 | 99.7 |
|  | 35 | 1 | .2 | .3 | 100.0 |
|  | Total | 288 | 64.3 | 100.0 |  |
| Missing | System | 160 | 35.7 |  |  |
| **Total** | | 448 | 100.0 |  |  |

| **Screening MRI CNS results** | | | | | |
| --- | --- | --- | --- | --- | --- |
|  | | Frequency | Percent | Valid Percent | Cumulative Percent |
| Valid | normal MRI | 260 | 58.0 | 83.9 | 83.9 |
|  | intraparenchymal melanosis only | 31 | 6.9 | 10.0 | 93.9 |
|  | other pathology | 19 | 4.2 | 6.1 | 100.0 |
|  | Total | 310 | 69.2 | 100.0 |  |
| Missing | System | 138 | 30.8 |  |  |
| **Total** | | 448 | 100.0 |  |  |
